# Restricted TCR Diversity and Expansion of CD177^+^CD8^+^ and TLR4^+^CD4^+^T Cell Subsets in Septic Shock‐Associated Immunosuppression: A Single‐Cell Multi‐Omics Study

**DOI:** 10.1002/mco2.70927

**Published:** 2026-08-17

**Authors:** Yawen Xie, Xunyao Wu, Guoyu Zhao, Xianli Lei, Jiahui Zhang, Na Cui, Bin Du

**Affiliations:** ^1^ Department of Critical Care Medicine Beijing Chao‐Yang Hospital Capital Medical University Beijing China; ^2^ State Key Laboratory of Complex Severe and Rare Diseases Clinical and Science Investigation Institute Peking Union Medical College Hospital Chinese Academy of Medical Science and Peking Union Medical College Beijing China; ^3^ Department of Critical Care Medicine Peking Union Medical College Hospital Chinese Academy of Medical Science and Peking Union Medical College Beijing China; ^4^ Medical ICU, Peking Union Medical College Hospital, Peking Union Medical College and Chinese Academy of Medical Sciences Beijing China

**Keywords:** CD8^+^ T, CD4^+^ T, septic shock‐associated immunosuppression, single‐cell RNA sequencing (scRNA‐seq), single‐cell T cell receptor sequencing (scTCR‐seq)

## Abstract

Sepsis‐induced immunosuppression contributes to poor outcomes in sepsis patients, yet the mechanisms underlying T cell dysfunction remain incompletely understood. We integrated single‐cell RNA sequencing (scRNA‐seq), paired single‐cell T cell receptor sequencing (scTCR‐seq), bulk RNA sequencing (RNA‐seq), and flow cytometry to characterize CD8^+^ and CD4^+^ T cells in septic shock‐associated immunosuppression. Patients showed reduced T cell receptor (TCR) clonotype diversity in CD4^+^ T cells but not CD8^+^ T cells, with restricted clonality correlating with disease severity and lymphopenia. Integrative analyses revealed the expansion of two disease‐associated subsets: (1) CD177^+^CD8^+^ T cells, enriched for interleukin‐17 (IL‐17) signaling with hyperexpanded clonotypes, and (2) TLR4^+^CD4^+^ T cells, enriched for phagocytosis‐ and lysosome‐related programs with restricted clonality. Both subsets expressed high levels of S100A8, S100A9, and S100A12, correlating positively with severity scores and systemic inflammation. These findings provided single‐cell resolution evidence that clonal restriction of CD4^+^ T cells is a hallmark of septic immunosuppression. Our study depicted a single‐cell atlas linking CD4^+^ clonal restriction and the expansion of pathogenic T cell subsets to outcome, highlighting TCR clonality as a biomarker of immune competence and CD177^+^CD8^+^ and TLR4^+^CD4^+^ T cells as candidate therapeutic targets in septic shock‐associated immunosuppression.

## Graphical abstract

Our study enrolled 10 septic shock patients with immunosuppression and 10 healthy controls and sorted CD8^+^ and CD4^+^ T cells for transcriptome sequencing. Disease‐specific cell types, key transcripts, and TCR features were identified using integrated analysis. Flow cytometry, western blotting, in vitro culture, and in vivo murine models were performed for verification.

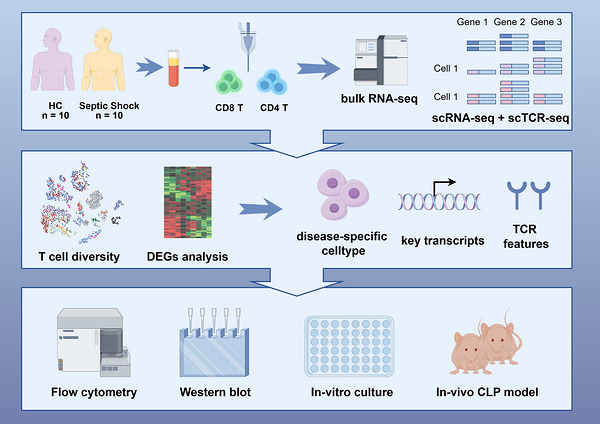

## 1 Introduction

Sepsis is a life‐threatening organ dysfunction caused by a dysregulated host response to infection, with high incidence and mortality. As the most severe subset of sepsis, septic shock is clinically diagnosed by persistent hypotension requiring vasopressor therapy to maintain a mean arterial pressure ≥ 65 mmHg, and a serum lactate level > 2 mmol/L despite adequate fluid resuscitation [1]. The in‐hospital mortality of patients with septic shock remains above 40%. Survivors are susceptible to secondary infections and physical dysfunction, with an elevated risk of long‐term mortality [2, 3]. A dysregulated immune response lies at the core of sepsis, yet the underlying mechanisms remain incompletely understood. Despite substantial progress in antimicrobial therapy and organ support in recent decades, mortality rates still remain high. Therefore, it is essential to further investigate the mechanisms of immune dysfunction in sepsis and septic shock.

The host response to sepsis is now characterized by concurrent hyperinflammation and immunosuppression. The immunosuppressive state develops early and often persists for weeks to months, contributing to secondary infections and late mortality. Sepsis‐associated immunosuppression (SAI) is characterized by impaired activation of both innate and adaptive immune responses. Its cellular hallmarks include lymphopenia, expansion of regulatory T cells (Tregs) and myeloid‐derived suppressor cells (MDSCs), reduced human leukocyte antigen‐DR (HLA‐DR) expression on monocytes, T cell dysfunction, and upregulation of inhibitory immune checkpoints [4, 5, 6]. Elucidating the mechanisms of sepsis‐induced immunosuppression is crucial for developing therapeutic strategies to restore immune function and improve outcomes [3, 7].

T cell dysfunction is a central feature of immune suppression in sepsis. During sepsis and septic shock, T cells exhibit impaired proliferation, cytokine production, and cytotoxic activity, predisposing the host to secondary infections. Mechanisms such as T cell exhaustion, marked by upregulation of inhibitory receptors like programmed death receptor 1 (PD‐1), cytotoxic T lymphocyte associated protein‐4 (CTLA‐4), T cell immunoglobulin and mucin‐domain containing‐3 (TIM‐3), and Treg cell expansion, further suppress immune responses [8, 9]. Increasing studies revealed marked heterogeneity of both CD4^+^ and CD8^+^ T cells by high‐resolution single‐cell RNA sequencing (scRNA‐seq) of peripheral blood mononuclear cells (PBMCs) from sepsis patients, including exhausted effector and immunoregulatory clusters correlating with disease severity and clinical outcomes [10, 11]. A recent multi‐omics study by Cao et al. profiled 281 adult and pediatric sepsis patients and identified several immune features, including an NR4A2^+^ exhausted CD4^+^ T cell subset [12]. However, how T cell repertoires and functional states are remodeled in septic shock‐associated immunosuppression remains incompletely characterized. Given the pivotal role of T cells in adaptive immune responses, understanding the dysregulation of T cell receptor (TCR) signaling in septic shock is critical for identifying therapeutic strategies to reverse T cell dysfunction.

TCR is essential for antigen recognition and T cell activation. In sepsis, impaired TCR signaling promotes T cell exhaustion and TCR downregulation, exacerbating immune dysfunction. This dysregulation shifts the immune response from hyperinflammation toward immune paralysis, further compromising T cell function. Restoring TCR‐mediated activation has emerged as a promising therapeutic strategy. Several clinical trials of immune checkpoint blockade (anti‐PD‐1 nivolumab and anti‐PD‐L1 BMS‐936559) and recombinant IL‐7 demonstrated the improvement of lymphopenia and T cell effector function in sepsis patients [13, 14].

This study aims to investigate TCR signaling in septic shock‐associated immunosuppression. By integrating scRNA‐seq, paired single‐cell TCR sequencing (scTCR‐seq), and RNA sequencing (RNA‐seq), we aim to characterize the heterogeneity of CD8^+^ and CD4^+^ T cells and their TCR repertoires, providing insights into T cell dysfunction and potential therapeutic targets.

## 2 Results

### 2.1 Profiling the T Cell Repertoire Landscape in Patients With Septic Shock‐Associated Immunosuppression

**FIGURE 1:**
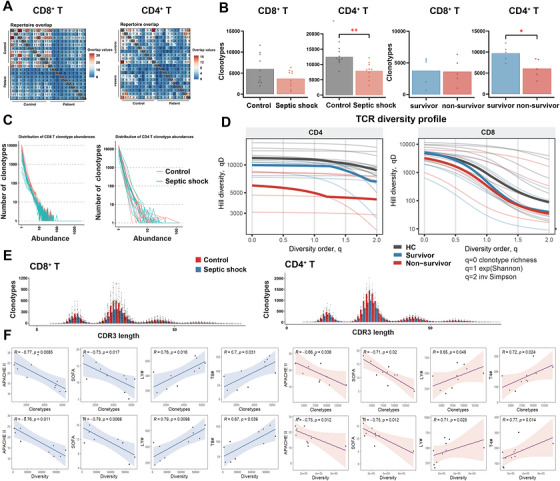
Reduced clonotype diversity of CD4^+^ T cells in septic patients with immunosuppression. (A) TCR repertoire overlapped across healthy controls (HCs; *n* =10) and patients with septic shock (*n* =10). (B) Comparison of unique TCR clonotypes between HCs (*n* =10) and patients with septic shock (*n* =10). (C) The distribution of clonotype abundance across all groups. (D) TCR diversity profiles (Hill numbers) of TCR repertoires. Hill diversity (qD) is plotted as a function of diversity order q (q = 0: clonotype richness, q = 1: the exponential of Shannon entropy; q = 2: inverse Simpson index). Thick lines indicate group means; thin lines represent individual samples. (HCs, *n* = 10; survivors, *n* = 5; non‐survivors, *n* = 5). E. The distribution of CDR3 number and length across all groups. (F) Correlation analysis of clonotype and diversity of CD8^+^ and CD4^+^ T cells with clinical variables (*n* = 10). *p < 0.05; **p < 0.01; ***p < 0.001; and ****p < 0.0001.

To map the clonality landscape of CD8^+^ and CD4^+^ T cells, we enrolled 10 septic shock patients with immunosuppression and 10 sex‐ and age‐matched healthy controls (HCs). PBMCs were collected, and CD8^+^ and CD4^+^ T cells were purified for T cell repertoire analysis. The clinical characteristics of septic shock patients with immunosuppression for sequencing were summarized in Table S1 and those of an additional validation cohort in Table S2. The purity of CD8^+^ and CD4^+^ T cells is shown in Figure S1. The repertoire overlaps of CD8^+^ and CD4^+^ T cells across all samples are illustrated in Figure 1A. Compared with HCs, septic shock patients with immunosuppression showed fewer TCR clonotypes in CD4^+^ T cells but not in CD8^+^ T cells, indicating a CD4^+^ repertoire contraction (Figure 1B). Among patients who were divided according to clinical outcomes, CD4^+^ T cell clonotypes were further reduced in non‐survivors, whereas CD8^+^ T cell clonotype number did not differ significantly between survivors and non‐survivors (Figure 1B). Overall distribution of clonotype abundances was similar between patients and HCs (Figure 1C), as were the number and length of CDR3 regions for CD8^+^ and CD4^+^ T cells (Figure 1E). To characterize TCR clonotype diversity more comprehensively, we profiled the repertoire across the full Hill‐number spectrum (q = 0 clonotype richness, q = 1 exp [Shannon], q = 2 inv Simpson). CD4^+^ T cell diversity was markedly reduced in sepsis patients and further declined in non‐survivors, whereas the CD8^+^ curves overlapped extensively among groups (Figure 1D). In addition, other diversity indicators were compared, including Chao1, Shannon, and inv Simpson. Consistently, CD4^+^T cell repertoire showed lower diversity in patients with unchanged CD8^+^T cell diversity, and a significant reduction in CD4^+^T cell diversity by the Shannon index (Figure S2). Inv Simpson index showed similar trend but without significance. Furthermore, both clonotype numbers and overall diversity correlated negatively with disease activity and positively correlated with the absolute lymphocyte and T cell counts (Figure 1F). Collectively, septic shock selectively impaired the clonotype diversity of CD4^+^ T cells, linking reduced repertoire diversity to disease severity and lymphopenia.

**FIGURE 2:**
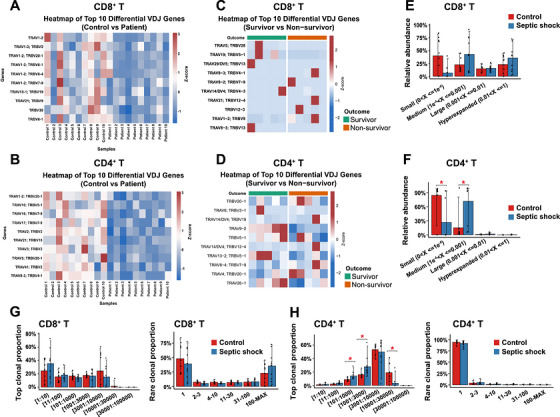
Preferential expansion of CD4^+^ TCR clonotypes in septic shock patients with immunosuppression. (A–B) The top 10 differential VDJ gene usages of CD8^+^ (A) and CD4^+^ (B) T cells in septic shock patients with immunosuppression (*n* = 10), compared to HCs (*n* = 10). (C–D) The top 10 differential VDJ gene usages of CD8^+^ (C) and CD4^+^ (D) T cells in non‐survivors (*n* = 10), compared to survivors (*n* = 10). (E–F) Relative abundance of TCR clonal expansion in septic shock patients with immunosuppression (*n* = 10) and HCs (*n* = 10). (G–H) Clonotype frequency distributions of CD8^+^ (G) and CD4^+^ (H) T cells from septic shock patients with immunosuppression (*n* = 10) and HCs (*n* = 10). *p < 0.05; **p < 0.01; ***p < 0.001; and ****p < 0.0001.

Building on these repertoire‐level alterations, we next investigated the preferential usage and clonal expansion. The top 10 differential VDJ genes usages of CD8^+^ and CD4^+^ T cells are shown in Figure 2A,B. Notably, the difference in CD8^+^ T cells was dominated by TRAV1‐2‐containing pairs (including the canonical TRAV1‐2/TRBV20‐1 and TRAV1‐2/TRBV6‐4), which were more frequent in HCs and most likely reflected the previously reported depletion of TRAV1‐2^+^ MAIT cells in sepsis, rather than remodeling of the conventional CD8^+^ repertoire [15]. The preferentially used V‐J gene pairs were also compared between survivors and non‐survivors (Figure 2C,D). At the level of clonal expansion, TCR clonotypes were more expanded in CD4^+^ T cells from patients, with a higher proportion of medium‐sized clonotypes than in HCs (Figure 2F,H). The expansion of CD4^+^ TCR clonotypes were concentrated in dominant rather than rare clonotypes. In contrast, CD8^+^ T cell clonal abundance did not differ significantly between patients and HCs (Figure 2E,G). Between survivors and non‐survivors, CD8^+^ clonotype distributions largely overlapped, whereas non‐survivors tended to have a higher proportion of medium and fewer small CD4^+^ clonotypes than survivors (Figure S3).

Considering the impact of age on TCR repertoire, we performed a subgroup analysis by age. Clonotype abundance and TCR diversity showed no correlation with age, and neither clonotype number, diversity, nor clonal expansion differed between patients aged ≤ 55‐ and > 55‐year‐old groups (Figure S4).

Overall, these findings indicate preferential expansion of CD4^+^ TCR clonotypes in septic patients with immunosuppression, whereas CD8^+^ clonotype, diversity, and clonal expansion distribution remain largely unaffected. Although clonal expansion was statistically comparable between survivors and non‐survivors, the V–J gene‐usage preferences were different.

### 2.2 Landscape of CD8^+^ and CD4^+^ T Cell Subsets and Their TCR Clonality in Septic Patients With Immunosuppression

**FIGURE 3:**
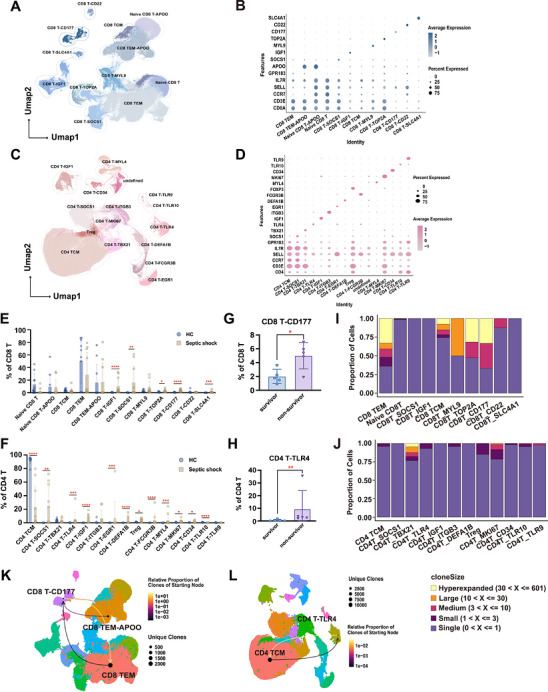
Altered peripheral CD8^+^ and CD4^+^ T cell subclusters and their TCR clonality in septic patients with immunosuppression. (A) Uniform Manifold Approximation and Projection (UMAP) representation of CD8^+^ T cells from septic patients with immunosuppression (*n* = 10) and healthy donor (*n* = 10) samples. Different colors indicate clusters. (B) Dot plot indicating average and percent expression of selected marker genes of CD8^+^ T cells. (C) UMAP representation of CD4^+^ T cells from septic shock (*n* = 10) and healthy donor samples (*n* = 10). Different colors indicate clusters. (D) Dot plot exhibiting average and percent expression of selected marker genes of CD4^+^ T cells. (E) Percentage analysis of CD8^+^ T cell subsets between patients with septic shock (*n* = 10) and healthy donors (*n* = 10). (F) Percentage analysis of CD4^+^ T cell subsets between patients with septic shock (*n* = 10) and healthy donors (*n* = 10). (G) Percentage analysis of CD8 T‐CD177 subset between survivor (*n* = 5) and non‐survivor (*n* = 5). (H) Percentage analysis of CD4 T‐TLR4 subset between survivor (*n* = 5) and non‐survivor (*n* = 5). Data are presented as mean ± SD. (I) Distribution of TCR clonotypes in 12 CD8^+^ T cell subtypes across all samples (*n* = 20). (J) Distribution of TCR clonotypes in 15 CD4^+^ T cell subtypes across all samples (*n* = 20). (K) Clonal network analysis of CD8^+^ T cell repertoires projected onto the UMAP. (L). Clonal network analysis of CD4^+^ T cell repertoires projected onto the UMAP. *p < 0.05; **p < 0.01; ***p < 0.001; and ****p < 0.0001.

To dissect the cellular impact of septic shock‐associated immunosuppression, we analyzed paired scRNA‐seq data generated from the same cDNA libraries. Unsupervised clustering of CD8^+^ T cells revealed 12 distinct subclusters, including naïve CD8 T (*CCR7*), naïve CD8 T‐APOO (*CCR7* and *APOO*), CD8 TCM (central memory T, *CCR7*, *SELL*, and *GPR183*), CD8 TEM (effector‐memory T, *CCR7*, *SELL*, *IL7R*, and *GPR183*), CD8 TEM‐APOO (*CCR7*, *SELL*, *IL7R*, *GPR183*, and *APOO*), CD8 T‐IGF1 (*IGF1*), CD8 T‐SOCS1 (*SOCS1*), CD8 T‐MYL9 (*MYL9*), CD8 T‐TOP2A (*TOP2A*), CD8 T‐CD177 (*CD177*), CD8 T‐CD22 (*CD22*), and CD8 T‐SLC4A1 (*SLC4A1*) annotated by marker gene expression (Figure 3A,B). Notably, five subclusters‐CD8 T‐IGF1, CD8 T‐SOCS1, CD8 T‐TOP2A, CD8 T‐CD177, and CD8 T‐SLC4A1 CD8^+^ T cell subclusters were significantly expanded in septic patients with immunosuppression, compared with HCs (Figure 3E). Among these, CD8 T‐CD177 expression was markedly lower in survivors than in non‐survivors (Figure 3G), whereas differences in the other CD8^+^ T cell subsets did not reach statistical significance (Figure S5). The proportion of each subset showed no correlation with age and was comparable between patients aged > 55 and ≤ 55 years (Figure S6).

Similarly, unsupervised clustering of CD4^+^ T cells identified 15 distinct subclusters, including CD4 TCM (*CCR7*, *SELL* and *GPR183*), CD4 T‐SOCS1 (*SOCS1*), CD4 T‐TBX21 (*TBX21*), CD4 T‐TLR4 (*TLR4*), CD4 T‐IGF1 (*IGF1*), CD4 T‐ITGB3 (*ITGB3*), CD4 T‐EGR1 (*EGR1*), CD4 T‐DEFA1B (*DEFA1B*), Treg (*FOXP3*), CD4 T‐FCGR3B (*FCGR3B*), CD4 T‐MYL4 (*MYL4*), CD4 T (*MKI67*), CD4 T (*CD34*), CD4 T‐TLR10 (*TLR10*), and CD4 T‐TLR9 (*TLR9;* Figure 3C,D). Most subclusters were significantly enriched in patients, compared with HCs, including CD4 T‐SOCS1, CD4 T‐TLR4, CD4 T‐IGF1, CD4 T‐EGR1, CD4 T‐DEFA1B, Treg, CD4 T‐FCGR3B, CD4 T‐MYL4, CD4 T‐MKI67, and CD4 T‐CD34 (Figure 3F). Importantly, CD4 T‐TLR4 exhibited a significantly higher proportion in non‐survivors than in survivors (Figure 3H), whereas other CD4^+^ T subsets showed no significant differences (Figure S5). As for the CD8^+^ subsets, the proportion of CD4 T‐TLR4 showed no correlation with age and was comparable between the > 55‐ and ≤55‐year groups (Figure S6).

Integrating scRNA‐ and scTCR‐seq data provided further insight into clonality. Approximately 30% of the CD8 T_CD177 subset was hyperexpanded (Figure 3I), and these clonotypes were extensively shared with the CD8 TEM and CD8 TEM‐APOO subsets (Figure 3K). In contrast, CD4 T_TLR4 clonotypes were predominantly small or single (Figure 3J), and were shared mainly with the CD4 TCM subset (Figure 3L). Taken together, these data reveal profound alterations in the peripheral CD8^+^ and CD4^+^ T cell compartments and their TCR clonality in septic shock patients with immunosuppression. In particular, CD8 T‐CD177 and CD4 T‐TLR4 displayed distinct patterns of expansion and clonal architecture, with differential associations with survival.

### 2.3 Markedly Increased CD177^+^ CD8 T Cells Enriched With Interleukin‐17 (IL‐17) Signaling Pathway

**FIGURE 4:**
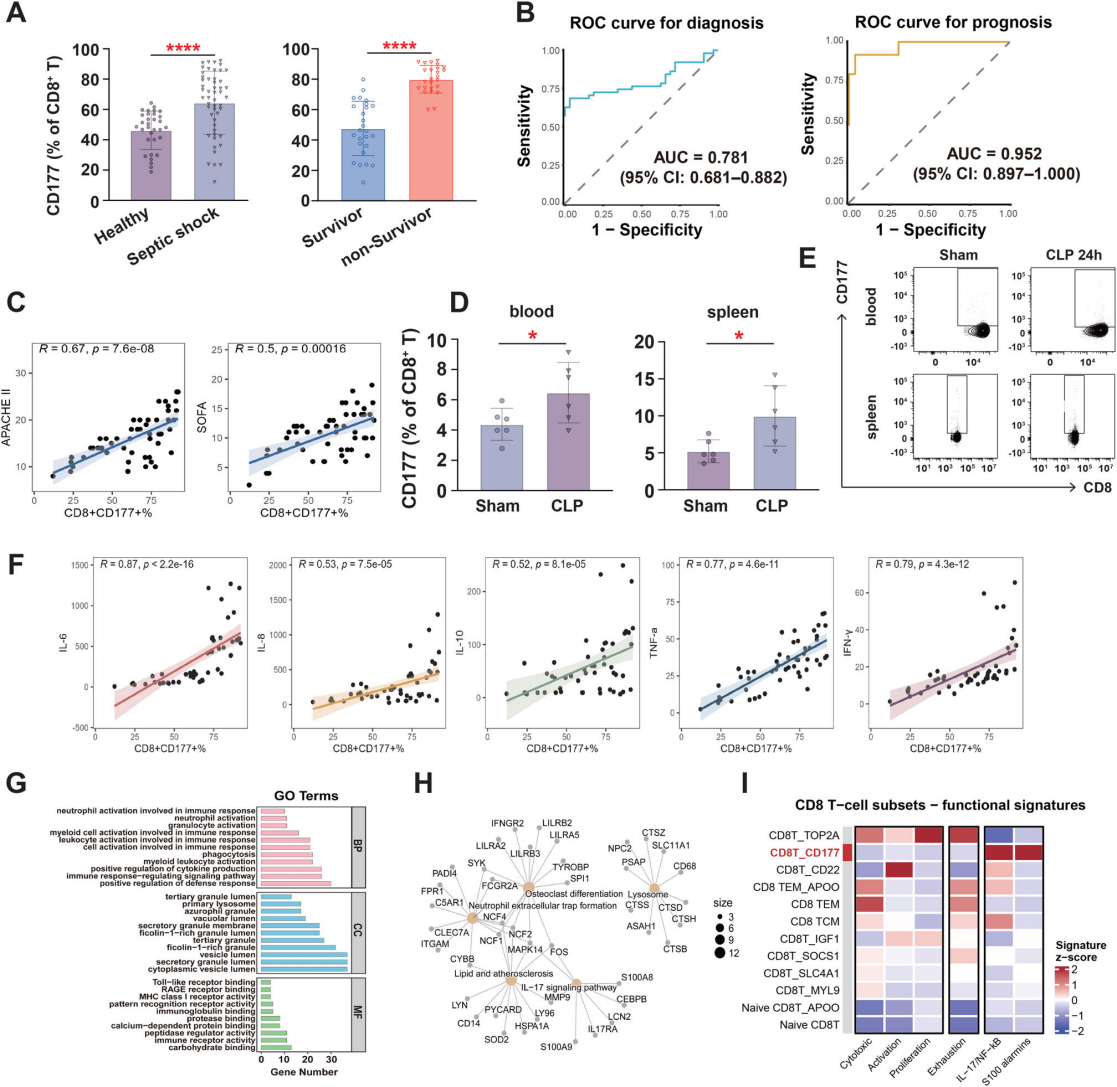
Identification of CD177^+^ CD8 T cell subtype in patients with septic shock. (A) Comparison of CD177 expression on CD8^+^ T cells between HCs (healthy, *n* = 32) and patients with septic shock (septic shock, *n* = 51) or survivor (*n* = 26) and non‐survivor patients (*n* = 25). (B) ROC curve for the proportion of CD177+CD8+T cells in distinguishing septic shock patients and predicting the prognosis (HCs, *n* = 32; patients, *n* = 51). (C) Scatter plot exhibiting the correlation between the CD177 expression on CD8^+^ T cells and APACHE II score and SOFA score (*n* = 51). (D‐E) Flow cytometry analysis of CD177 expression on CD8^+^ T cells from the blood and spleen in sham (*n* = 6) or CLP mice (*n* = 6) at 24 h. (F) Scatter plot exhibiting the correlation between the CD177 expression on CD8^+^ T cells and serum IL‐6, IL‐8, IL‐10, TNF‐α, and IFN‐γ (*n* = 51). (G) Bar charts exhibiting top 10 GO terms enriched in upregulated genes of CD8 T‐CD177 subset. (H) Network graph listed the enriched pathways in the CD8 T‐CD177 subset by KEGG analysis. (I) Heatmap of functional gene‐signature scores of CD8^+^ T cell subsets. Data are presented as mean ± SD. *p < 0.05; **p < 0.01; ***p < 0.001; and ****p < 0.0001.

Given the preferential expansion of CD8 T–CD177 cells identified above, we next validated their expression and functional characteristics. Flow cytometry confirmed higher CD177 expression on total CD8^+^ T cells in patients, compared with HCs, with expression particularly elevated in non‐survivors (Figure 4A). The proportions of total T cells and CD8^+^T cells were also remarkably reduced in patients, compared with HCs, although neither differed significantly between survivors and non‐survivors (Figure S7).

ROC analysis demonstrated the clinical value of CD177 expression on CD8^+^T cells as a biomarker, with an AUC of 0.781 (95% CI, 0.681–0.882) for diagnosis and 0.952 (95% CI, 0.897–1.000) for predicting mortality among septic shock patients with immunosuppression (Figure 4B). Gating strategy was shown in Figure S8. CD177 expression positively correlated with disease activity (acute physiology and chronic health evaluation II [APACHE II] and sequential organ failure assessment [SOFA] score) and with inflammatory status (serum IL‐6, IL‐8, IL‐10, TNF‐α, and IFN‐γ; Figure 4C,F). Consistently, sepsis‐surviving mice following cecal ligation and puncture (CLP) showed increased CD177 expression on CD8^+^ T cells in both blood and spleen, compared with sham mice (Figure 4D,E).

Gene ontology (GO) analysis of upregulated genes in CD177^+^CD8^+^ T cells revealed enrichment for cytoplasmic vesicle lumen, secretory granule lumen, vesicle lumen, and positive regulation of defense response (Figure 4G). Kyoto encyclopedia of genes and genomes (KEGG) enrichment analysis linked these upregulated genes to the lysosome and IL‐17 signaling pathways (Figure 4H). To characterize the functional properties of the CD8^+^ T cell subsets, we scored each subset for functional gene signatures using AUCell. The CD177^+^CD8^+^T cell subset was selectively enriched for the IL‐17/NF‐κB and S100‐alarmin signatures, with minimal enrichment for the canonical cytotoxicity, activation, proliferation, and exhaustion programs (Figure 4I). This profile clearly distinguished CD8 T‐*CD177* from classical effector‐memory CD8^+^ T cells and from the proliferating CD8 T‐*TOP2A* subset, indicating that these cells are not conventional cytotoxic or terminally exhausted effectors but a transcriptionally distinct, alarmin‐producing population with active IL‐17 signaling. These results were concordant with the GO and KEGG enrichment and with the elevated S100A8/A9/A12 expression validated below.

**FIGURE 5:**
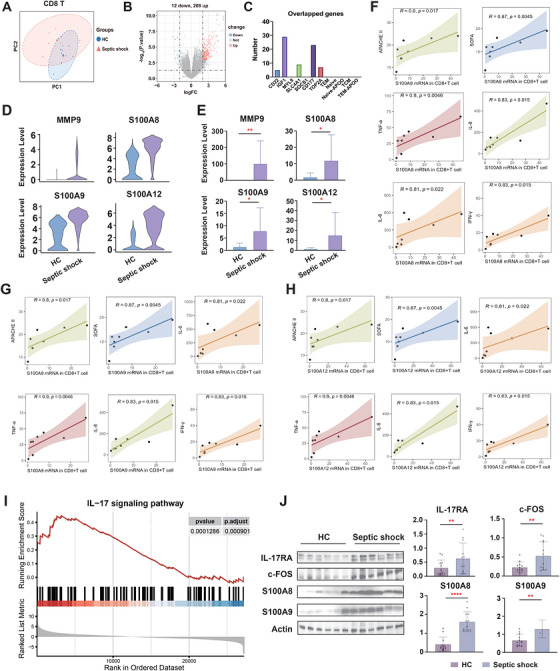
Identification of feature genes and signaling pathways of CD177^+^ CD8 T cell subtype in patients with septic shock. (A) PCA plot of bulk RNA‐seq data in HCs (*n* = 10) and patients with septic shock (*n* = 8). (B) The volcano plot exhibited 220 significant DEGs between HCs (*n* = 10) and septic groups (*n* = 8). Significantly increased (logFC > 2 and *p*‐value < 0.05) genes are highlighted in red, and significantly decreased (logFC < −2 and *p*‐value < 0.05) genes are highlighted in blue. (C) Upregulated DEGs were intersected with featured gene signatures of 12 CD8^+^ T cell subtypes, and the intersected numbers are illustrated in bar charts. (D) Violon plot revealing the expressions of *CD177*, *MMP9*, *S100P*, *S100A8*, *S100A9*, and *S100A12* in the CD8 T‐CD177 subset of HCs (*n* = 10) and patients with septic shock (*n* = 10). (E) Comparison of *CD177*, *MMP9*, *S100P*, *S100A8*, *S100A9*, and *S100A12* expressions in CD8 T cells of HCs (*n* = 8) and patients with septic shock (*n* = 8) by real‐time PCR. (F) Scatter plot exhibiting the correlation between the S100A8 expression in CD8^+^ T cells and APACHE II score, SOFA score, serum IL‐6, IL‐8, TNF‐α, and IFN‐γ (*n* = 8). (G) Scatter plot revealing the correlation between the S100A9 expression in CD8^+^ T cells and APACHE II score, SOFA score, serum IL‐6, IL‐8, TNF‐α, and IFN‐γ (*n* = 8). (H) Scatter plot demonstrating the correlation between the S100A12 expression in CD8^+^ T cells and APACHE II score, SOFA score, serum IL‐6, IL‐8, TNF‐α, and IFN‐γ (*n* = 8). (I) GSEA enrichment plot for IL‐17 signaling pathway in the bulk transcriptome of CD8^+^T cells from septic shock patients (*n* = 8), compared with HCs (*n* = 8). (J) Validation of IL‐17 signaling pathway‐related proteins by western blotting in CD8^+^ T cells of HCs (*n* = 12) and patients with septic shock (*n* = 12). Each plot represents a dependent individual, and data are presented as mean ± SD. *p < 0.05; **p < 0.01; ***p < 0.001; and ****p < 0.0001.

To link this subset to transcriptomic changes, we integrated bulk RNA‐seq and scRNA‐seq dataset. The principal component analysis (PCA) plot demonstrated two sample clusters in the HC and septic groups (Figure 5A). Ranking all expressed genes by the mean expression in patient CD8^+^ T cells revealed a bimodal distribution, in which CD177 was in the higher‐expression mode (Figure S9), indicating that CD177 is a moderately expressed, sepsis‐induced gene. There were 220 differential expressed genes (DEGs) in the septic group, compared to HCs, including 208 upregulated and 12 downregulated genes (Figure 5B). Intersecting the upregulated DEGs with the signature genes of the 12 CD8^+^ subsets identified 23 genes that overlapped with the CD177^+^CD8^+^ signature (Figure 5C). *MMP9*, *S100A8*, *S100A9*, and *S100A12* expressions were also higher in patient‐derived CD177^+^ CD8 T cells, compared to those in HCs (Figure 5D), and their upregulation in total CD8^+^ T cells was confirmed by RT‐polymerase chain reaction (PCR; Figure 5E). Furthermore, correlation analysis revealed that S100A8, S100A9, and S100A12 expressions in total CD8^+^ T cells were positively correlated with APACHEII, SOFA score, and serum IL‐6, IL‐8, TNF‐α, and IFN‐γ (Figure 5F–H).

To validate the IL‐17 signal activation at the transcriptomic level, we performed GSEA on the RNA‐seq of CD8^+^ T cells. The IL‐17 signaling pathway was significantly and positively enriched in patients (Figure 5I), consistent with the IL‐17/NF‐κB activation identified in CD177^+^CD8^+^ T cells at single‐cell resolution. We further performed an immunoblotting assay and confirmed higher IL‐17RA, c‐FOS, S100A8, and S100A9 protein expression in patient‐derived CD8^+^ T cells, compared with HCs (Figure 5J). Besides, we further investigated whether the transcription of *S100A8*, *S100A9*, and *S100A12* in T cells was related to their serum levels. Serum S100A8, S100A9, and S100A12 were markedly higher in patients than in HCs (Figure S10), and further elevated in non‐survivors, compared with survivors (Figure S10). Moreover, the transcriptional levels of S100A8, S100A9, and S100A12 in CD8^+^ T cells were positively correlated with their corresponding serum concentrations (Figure S10). Consequently, the above results implicated an expansion of CD177^+^CD8^+^ T cells enriched with the IL‐17 signaling in patients with septic shock.

### 2.4 Enrichment of TLR4^+^ CD4 T Cells in Septic Patients With Immunosuppression

**FIGURE 6:**
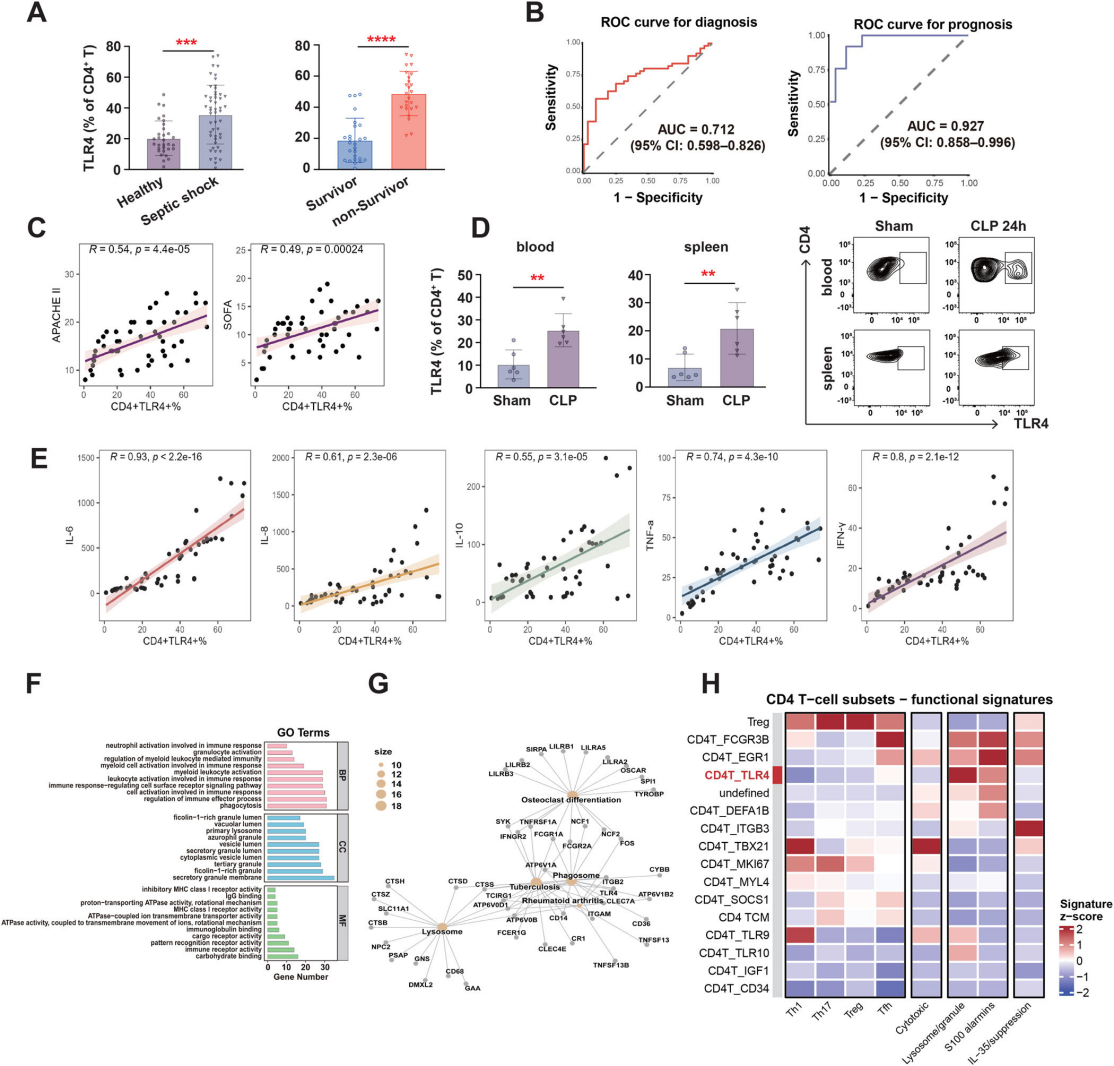
Identification of TLR4^+^ CD4 T cell subtype in patients with septic shock. (A) Comparison of the TLR4 expression on CD4^+^ T cells between HCs (healthy, *n* = 32) and patients with septic shock (septic shock, *n* = 51) or survivor (*n* = 26) and non‐survivor patients (*n* = 25). (B) ROC curve of TLR4+CD4+ T cell proportion for distinguishing septic shock patients and predicting prognosis (HCs, *n* = 32; patients, *n* = 51). (C) Correlation between the TLR4 expression on CD4^+^ T cells and APACHE II score and SOFA score (*n* = 51). (D) Flow cytometry analysis of the TLR4 expression on CD4^+^ T cells from blood and spleen in sham (*n* = 6) or CLP (*n* = 6) mice at 24 h. (E) Correlation between the proportion of TLR4^+^CD4^+^ T cells and IL‐6, IL‐8, IL‐10, IFN‐γ, and TNF‐α (*n* = 51). F. Bar charts of the top 10 GO terms enriched in the upregulated genes of CD4 T‐TLR4 subset. (G) Network graph listed the enriched pathways in CD4 T‐TLR4 subset by KEGG analysis. (I) Heatmap of functional gene‐signature scores of CD4^+^ T cell subsets. Data are presented as mean ± SD. *p < 0.05; **p < 0.01; ***p < 0.001; and ****p < 0.0001.

In parallel with CD8 T‐CD177, we explored the significance of CD4 T‐TLR4. Flow cytometry confirmed higher TLR4 expression on CD4^+^ T cells in patients than in HCs, with higher levels in non‐survivors (Figure 6A). The proportions of total T cells and CD4^+^ T cells were markedly reduced in patients relative to HCs but neither differed significantly between survivors and non‐survivors (Figure S11). ROC curve analysis supported the value of CD4^+^ T cell TLR4 expression as a biomarker, yielding an AUC of 0.712 (95% CI, 0.598–0.826) for diagnosis and 0.927 (95% CI: 0.858–0.996) for predicting mortality among patients with septic shock‐associated immunosuppression (Figure 6B). Gating strategy was displayed in Figure S12. TLR4 expression correlated positively with disease severity and inflammatory status (Figure 6C,E). Consistently, CLP‐surviving mice showed increased TLR4 expression on CD4+ T cells, compared with sham mice (Figure 6D).

GO analysis of the genes upregulated in TLR4+CD4+ T cells revealed enrichment for secretory granule membrane, ficolin‐1‐rich granule, phagocytosis, and regulation of immune effector processes (Figure 6F), and KEGG analysis correlated these genes to the lysosome and phagosome pathways (Figure 6G). To characterize the CD4^+^ subset function, we applied the AUCell signature‐scoring approach. The TLR4^+^CD4^+^T cell subset was selectively enriched for the lysosome/granule and S100‐alarmin signatures, with minimal enrichment for the canonical helper and regulatory functions, including Th1, Th17, Treg, Tfh, cytotoxic, and IL‐35/suppression signatures (Figure 6H). This profile distinguished TLR4^+^CD4^+^T cells from classical helper and regulatory subsets, indicating that they are not conventional helper or suppressive cells but a transcriptionally distinct, alarmin‐producing population oriented toward lysosomal and phagocytic activity, consistent with the GO and KEGG results and with the elevated S100A8/A9 expression.

**FIGURE 7:**
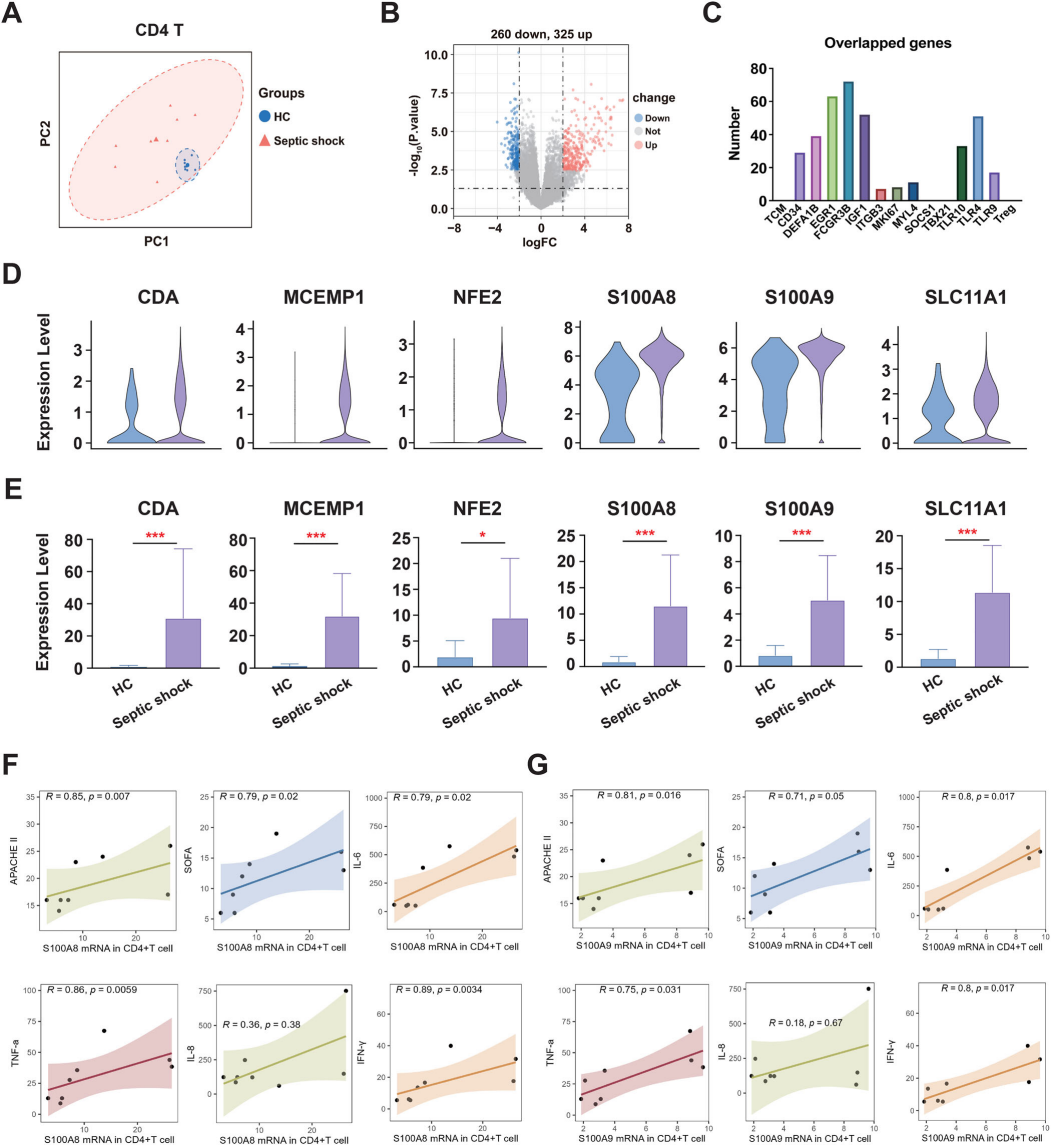
Identification of feature genes of TLR4^+^ CD4 T cell subtype in patients with septic shock. (A) PCA plot of bulk RNA‐seq data in HCs (*n* = 10) and patients with septic shock (*n* = 8). (B) The volcano plot depicted 585 significant DEGs between HCs (*n* = 10) and septic groups (*n* = 8). Significantly increased (logFC > 2 and *p*‐value < 0.05) genes are highlighted in red, and significantly decreased (logFC < −2 and *p*‐value < 0.05) genes are highlighted in blue. (C) Upregulated DEGs were intersected with featured gene signatures of 15 CD4^+^ T cell subtypes, and the intersected numbers are illustrated in bar charts. (D) Violin plot exhibiting the expressions of *CDA*, *MCEMP1*, *NFE2*, *S100A8*, *S100A9*, and *SLC11A1* in CD4 T‐TLR4 subset of HCs (*n* = 10) and patients with septic shock (*n* = 10). (E) Comparisons of *CDA*, *MCEMP1*, *NFE2*, *S100A8*, *S100A9*, and *SLC11A1* expressions in CD4 T cells of HCs (*n* = 8) and patients with septic shock (*n* = 8) by real‐time PCR. (F) Scatter plot exhibiting the correlation between the S100A8 expression in CD4^+^ T cells and APACHE II score, SOFA score, serum IL‐6, IL‐8, TNF‐α, and IFN‐γ (*n* = 8). (G) Scatter plot depicting the correlation between the S100A9 expression in CD4^+^ T cells and APACHE II score, SOFA score, serum IL‐6, IL‐8, TNF‐α, and IFN‐γ (*n* = 8). Data are presented as mean ± SD. *p < 0.05; **p < 0.01; ***p < 0.001; and ****p < 0.0001.

We then analyzed bulk RNA‐seq data from total CD4^+^ T cells of patients and matched HCs. PCA separated the patient and HC samples (Figure 7A). The volcano plot identified 325 upregulated and 260 downregulated genes in patient CD4^+^ T cells relative to HCs (Figure 7B). Intersecting the upregulated genes with the signature genes of the 15 CD4^+^ subsets identified 51 genes that overlapped with the TLR4^+^CD4^+^ signature (Figure 7C). The expressions of *CDA*, *MCEMP1*, *NFE2*, *S100A8*, *S100A9*, and *SLC11A1* were higher in patient‐derived TLR4^+^ CD4 T cells, compared to those from HCs (Figure 7D), and their upregulation in total CD4+ T cells was confirmed by RT‐PCR (Figure 7E). S100A8 and S100A9 expression in total CD4^+^ T cells positively correlated with disease activity and serum inflammatory cytokines (Figure 7F,G). The transcriptional levels of S100A8 and S100A9 in CD4^+^ T cells were also positively correlated with their serum concentrations (Figure S13). Together, these results implicate TLR4^+^CD4^+^ T cells as a functionally distinct subset in septic shock‐associated immunosuppression that is closely associated with disease progression and inflammatory burden.

## 3 Discussion

In this study, we applied multi‐omics approaches to delineate the transcriptional programs and TCR clonotypes of CD8^+^ and CD4^+^ T cells at single‐cell resolution in patients with septic shock‐associated immunosuppression. We identified a clonally expanded CD177^+^CD8^+^ T cell subset enriched for IL‐17 signaling, and a TLR4^+^CD4^+^ T cell subset enriched for phagocytosis‐ and lysosome‐related pathways. Importantly, both subsets were more abundant in non‐survivors and closely associated with disease severity and systemic inflammation, suggesting that they may contribute to the dysregulated immune response in sepsis. These findings extend our understanding of the immune landscape in patients with septic shock‐associated immunosuppression, highlighting the contributions of skewed TCR clonality and altered effector programs.

A central and novel aspect of our study is the detailed characterization of the TCR repertoire in patients with septic shock‐associated immunosuppression. Previous studies have provided preliminary evidence that reduced TCR diversity is a hallmark of sepsis‐induced immune dysfunction. Venet et al. first demonstrated a decrease in overall TCR repertoire diversity in sepsis patients, compared with HCs, while Tomino et al. reported that restricted TCR diversity, together with elevated PD‐1 expression, predicted mortality in patients with septic shock [16, 17]. Our findings build upon these observations: whereas CD8^+^ T cells displayed relatively preserved diversity, CD4^+^ T cells from patients with sepsis‐induced immunosuppression showed a striking reduction in clonotypic diversity and preferential expansion of medium‐sized and dominant clonotypes. This restricted CD4^+^ TCR landscape may underlie impaired antigen recognition and insufficient adaptive responses, thereby contributing to sustained immunosuppression.

Several previous studies have indicated that the IL‐17 signaling pathway is key to sepsis. Single‐cell analysis and bulk‐RNA‐seq of blood samples from patients with sepsis revealed that DEGs were significantly enriched in the IL‐17 signaling pathway in T cells between HCs and patients with sepsis. Moreover, IL‐17 signaling pathway‐associated genes, including *IL‐17R*, *TRAF6*, *RELB*, *TRAF5*, *CEBPB*, *JUNB*, *CXCL1*, *CXCL3*, *CXCL8*, *CXCR1*, and *CXCR2*, were significantly upregulated in sepsis blood samples than HCs [18]. Elevated levels of IL‐17 were found in the serum of patients with sepsis early after sepsis onset and were proposed as diagnostic biomarkers [19]. Mechanistically, IL‐17A could activate NLRP3 inflammasome and induce pyroptosis of alveolar epithelial cells, contributing to sepsis‐induced acute lung injury [18, 20, 21]. In a mouse model of sepsis‐associated encephalopathy, blocking the IL‐17A/IL‐17R pathway inhibited microglial activation, neuroinflammation, and partially reversed sepsis‐induced cognitive impairment [22]. Our single‐cell analysis identified a CD177+CD8+ T cell subset that was more abundant in both clinical and murine sepsis, correlated with disease severity and inflammatory status, and was enriched for IL‐17 signaling. Further mechanism research into the role of IL‐17 signaling pathway in CD177^+^CD8^+^T cells is warranted and may reveal more therapeutic targets for sepsis immunotherapy.

In addition, the hyperexpanded CD177^+^CD8^+^ clonotypes were clonally shared with effector‐memory CD8^+^ T cells. As this clonal‐network analysis reflected clonal relationships rather than a differentiation trajectory, the direction cannot be inferred from the network alone. Nevertheless, the CD177^+^CD8^+^ subset was transcriptionally distinct from conventional effector‐memory cells, being enriched for IL‐17/NF‐κB and S100‐alarmin programs while showing minimal enrichment for cytotoxic, proliferative, exhaustion, and stem‐/memory‐like signatures. The presence of this activated effector‐skewed profile with marked clonal expansion of CD177^+^ subset in septic shock is most consistent with an antigen‐independent activation of pre‐existing effector‐memory CD8^+^ T cells, a process known as bystander activation [23]. Memory T cells are stimulated by systemic inflammatory cytokines (such as IL‐12, IL‐15, and IL‐18) in the absence of antigens. Notably, the existence of bystander activation in sepsis has been demonstrated by Serbanescu et al., which indicated that memory CD8^+^ T cells acquired an activated phenotype independent of TCR engagement, most likely through the role of cytokines [24]. Therefore, considering that these clonally related effector‐memory cells may derive from the pre‐existing memory pool, it is possible that the immuno‐environment characterized by various cytokines could drive the rapid transition into the CD177^+^ subset rather than requiring the time‐consuming differentiation of new memory cells. The role of CD177^+^CD8^+^ T cells in septic shock and associated multiple organ failure warrants further investigation.

We also identified a TLR4^+^CD4^+^ T cell subset in patients with septic shock, whose clonotypes were shared mainly with central memory CD4^+^ T cells and which may likewise arise from this pre‐existing memory population. Notably, *S100A8*, *S100A9*, and *S100A12* expression were higher in patient‐derived CD177^+^CD8^+^ and TLR4^+^CD4^+^ T cells, compared to those in HCs. S100A8, S100A9, and S100A12 belong to the family of calgranulins, which can appear as a heterodimer known as S100A8/A9 or calprotectin, and S100A12 (calgranulin C) [25]. Plasma levels of S100A8/S100A9 and S100A12 were higher in patients with septic shock than HCs, particularly higher in non‐survivors than in survivors [26]. S100A8/A9 and S100A12 acted as a pathological player in sepsis development and consequential organ damage. S100A8/A9 contributed to sepsis‐induced cardiomyopathy by promoting mitochondrial dysfunction, and its blockade in a mouse model of endotoxemia exerts potent anti‐inflammatory effects and mitigates myocardial dysfunction [27, 28]. S100A8/A9 reduced the survival rate, aggravated pulmonary inflammation, acute liver injury, neuroinflammation, and kidney injury in the murine septic model [29, 30, 31, 32, 33, 34, 35, 36, 37, 38]. S100A12 could competitively bind to TLR4 and receptor for advanced glycation end products (RAGE), decreasing IL‐10 and TNF‐α production, and might be associated with sepsis‐related mortality [39, 40]. Serum S100A12 is elevated in patients with sepsis‐induced acute respiratory distress syndrome (ARDS) and in septic mice, and S100A12 induces the secretion of inflammatory cytokines, chemokines, and cell adhesion molecules [41]. In our study, S100A8/A9 and S100A12 expression in CD8^+^ T cells and S100A8/A9 expression in CD4^+^ T cells from patients correlated positively with disease severity and serum inflammatory cytokines, suggesting that CD177^+^CD8^+^ and TLR4^+^CD4^+^ T cells may promote septic shock through S100A8/A9 and S100A12 secretion. Accordingly, regulating the production and function of S100A8/A9 and S100A12 in T cells may represent a candidate immunotherapeutic strategy for sepsis.

Given that S100A8, S100A9, and S100A12 are predominantly myeloid‐derived, the expression in CD177^+^CD8^+^ and TLR4^+^CD4^+^ T cells is particularly noteworthy. Together with other granule‐ and myeloid‐associated genes enriched in these subsets (such as *MMP9*, *MCEMP1*, and *SLC11A1*) and the selective increase in the S100‐alarmin signature, these findings indicated that certain T cell subsets were characterized by a myeloid‐like phenotype with S100‐alarmin production during septic shock. Besides, as the endogenous ligand of TLR4 and RAGE, increased S100A8/A9 and S100A12 likely converge on NF‐κB signaling [42]. TLR4^+^CD4^+^ T cells co‐expressing S100A8/A9 and their receptors can sustain self‐amplifying feedback loop, whereas NF‐κB activation driven by S100A8/A9 and S100A12 may reciprocally activate IL‐17 signaling pathway in CD177^+^CD8^+^ T cells, which provides a plausible explanation for how depleted T cells could still sustain persistent inflammation in septic shock‐induced immunosuppression. In addition, an HLA‐DR^low^ S100A^high^ monocyte subset has been shown to be immunosuppressive and to restrain CD4^+^ T cell proliferation [43]. In our study, the S100A‐expressing T cells remained functionally active and pro‐inflammatory, supporting the view that sepsis‐induced immunosuppression is not a uniform suppression of immune cells but a coexistence of suppressed and aberrantly activated populations. Given the growing recognition of S100A8/A9 and S100A12 as both drivers of sepsis‐associated organ injury and as biomarkers [44, 45], these data further support targeting the S100–TLR4/RAGE axis to dampen both systemic inflammation and the aberrant T cell programs.

More intriguingly, in spite of enrolled patients with septic shock‐associated immunosuppression defined by absolute lymphocyte count < 1000 /µL, our results indicated that the proportions of CD177^+^CD8^+^ and TLR4^+^CD4^+^ T cells were increased and were closely correlated with disease severity and clinical outcome. Moreover, increased expression of S100A8/9/12 and activation of the IL‐17 signaling pathway suggested that both TLR4^+^CD4^+^ T cells and CD177^+^CD8^+^ were functionally active. It is widely accepted that patients with sepsis‐induced immunosuppression have a decreased immune cell number and impaired cell function as well. Consequently, immunostimulatory therapies have been attempted to restore and activate the compromised immune function [46]. Nevertheless, clinical trials have so far failed to confirmed that immunostimulatory therapies improve the prognosis of immunosuppressed septic patients [47, 48], even though several preclinical researches in vivo have shown that immunostimulants such as thymosin α1 and IL‐7 alleviated sepsis‐induced immunosuppression [49, 50]. Notably, our study provides novel evidence that in the immunosuppressed septic population characterized by a decreased number of lymphocytes, the proportions of TLR4^+^CD4^+^ T cells and CD177^+^CD8^+^ T cells were increased with functional activation, indicating that the mechanism of sepsis‐induced immunosuppression is complex. Even if the number of immune cells decreases, their immune function is not necessarily inhibited. From the perspective of restoring cellular immunity, merely stimulating T cell activation is probably not suitable for all sepsis‐induced immunosuppression patients. This may also partially explain the limited efficacy of immunostimulatory therapy and underscores that elucidating the cellular mechanisms of sepsis‐induced immunosuppression is essential for developing clinically effective treatments.

CD177^+^CD8^+^ and TLR4^+^CD4^+^ T cells represent potential candidates for therapeutic targets in sepsis. First, the two subsets were functionally distinct populations converging on alarmin production, together with IL‐17/NF‐κB signaling in CD177^+^CD8^+^ cells and lysosomal/phagocytic activity in TLR4^+^CD4^+^ cells, rather than on conventional effector, helper, or regulatory function. Second, the proportions of both subsets correlated positively with circulating pro‐inflammatory cytokines such as IL‐6 and TNF‐α, positioning them as pro‐inflammatory effectors that sustained the dysregulated inflammatory response. Third, their proportions had diagnostic value for identifying septic shock and prognostic value for mortality, suggesting their utility as biomarkers for immune monitoring and outcome prediction. Finally, the effector pathways enriched in these cells could be pharmacologically targeted. S100A8/A9 can be inhibited with small molecules such as ABR‐238901, the IL‐17/IL‐17RA axis can be neutralized with anti‐IL‐17 or anti‐IL‐17R biologics, and TLR4 signaling can be blocked with antagonists such as TAK‐242 [51, 52]. Preclinical studies showed that S100A8/A9 blockade attenuated sepsis‐associated organ injury [28, 53]. Collectively, these features nominated CD177^+^CD8^+^ and TLR4^+^CD4^+^ T cells as candidate targets for precision immunomodulation, complementing conventional immunostimulatory strategies in septic shock‐associated immunosuppression.

The proportion of CD177^+^CD8^+^ T cells measured by flow cytometry exceeded both the size of the transcriptome‐defined CD8 T‐CD177 cluster and the CD177^+^ fraction in the CLP model. A cluster defined by scRNA‐seq is not equivalent to a surface‐marker positivity rate because cluster assignment depends on the overall transcriptome rather than on CD177 alone. In addition, transcript and protein levels are often discordant. mRNA abundance explains only about 40% of the variance in protein levels, and protein abundance is controlled predominantly at the translational level [54, 55]. In droplet‐based scRNA‐seq, moderately expressed transcripts are frequently observed as zeros in individual cells as a consequence of limited capture and limited sequencing depth [56]. A moderately expressed, sepsis‐induced surface protein such as CD177 can therefore be broadly detected by flow cytometry while appearing sparse at the transcript level. The lower CD177^+^ proportion in mice likewise results from cross‐species differences in antibody epitopes, baseline CD177 biology, and disease stage. Taken together, the bulk, single‐cell, flow‐cytometric, and murine data are mutually consistent once these methodological differences are accounted for.

However, our study has several limitations. First, the sample size for RNA‐seq was relatively small, comprising only 10 patients and 10 HCs, which limited its statistical power. Although the core findings were validated in an independent cohort of 83 participants, the modest sample size may still limit the robustness of sequencing results, and further validation in larger cohorts is required to confirm the clinical implications of these findings. Second, sepsis patients are intrinsically heterogeneous. Several biomarkers are available to assess immune status in sepsis patients, such as HLA‐DR expression. Our research focused on septic shock patients with immunosuppression and adopted the absolute lymphocyte count < 1000 /µL as the inclusion criterion. Despite its relatively simplicity, the indicator is widely accessible in clinical practice and is recommended in relevant consensus for defining immunosuppression [43, 57]. It is therefore expected to serve as a reliable and reproducible criterion for future clinical studies. All samples were collected at a single time point in the study. Given that immune status changes dynamically over the course of sepsis, longitudinal studies with serial sampling are needed to characterize the temporal dynamics of CD177^+^CD8^+^ and TLR4^+^CD4^+^ T cells and to further elucidate their roles in the immune assessment and potential treatment of SAI. In addition, our analyses were restricted to peripheral blood and therefore cannot capture the characteristics of tissue‐ and organ‐resident T cells, which warrants further investigations. Furthermore, our study focused primarily on transcriptomic analysis, without in vitro functional assays and in vivo loss‐of‐function experiments. To partially address this issue, we established a validation cohort and constructed the CLP mouse model, which confirmed the existence and clinical value of the two T cell subsets. In follow‐up work, we will conduct more mechanistic and functional studies and targeted intervention experiments to address the current limitations. Together, these efforts will provide a solid foundation for further investigation of sepsis‐induced immunosuppression and the identification of potential therapeutic targets.

In summary, we provide a multi‐omics atlas of T cell subsets and TCR clonality in patients with septic shock‐associated immunosuppression. By highlighting clonally restricted CD4+ T cells and identifying disease‐associated subsets such as CD177^+^CD8^+^ and TLR4^+^CD4^+^ T cells, our study advances the understanding of immune dysregulation in sepsis. These results not only reinforce the central role of impaired TCR repertoire diversity in sepsis‐induced immunosuppression but also uncover novel therapeutic targets that may inform future strategies for restoring immune competence and improving clinical outcomes.

## 4 Materials and Methods

### 4.1 Patients and Ethics

This prospective study was performed at Peking Union Medical College Hospital from January 2024 to September 2024. We screened patients admitted to ICU daily and enrolled patients with SAI following the inclusion criteria: (1) Aged between 18 and 70; (2) met the diagnosis of septic shock according to sepsis 3.0 definition within 24 h on admission [1]; (3) absolute lymphocyte count < 1000 /µL according to 2022 SAI expert consensus and our previous researches [57, 58, 59]; (4) the minimum dose of norepinephrine more than 0.1 µg/kg/min; (5) requirement for mechanical ventilation. The diagnosis of SAI was confirmed independently by two experienced physicians. The exclusion criteria included: (1) during pregnancy or lactation, (2) malignant tumor or hematological diseases, (3) autoimmune diseases, (4) receiving high‐dose glucocorticoid (over 300 mg/day of hydrocortisone or 15 mg/day of cortisone) or other immunosuppressants, (5) HIV infection, and (6) viral hepatitis.

Informed consent was obtained from the participants or their authorized relatives. The study obtained approval from the Ethics Committee of the Peking Union Medical College Hospital (Approval No. I‐22PJ1104).

### 4.2 Murine Sepsis Model

C57BL/6J male mice (6–8 weeks old) were purchased from Viewsolid Biotech (Beijing, China) and raised under specific pathogen‐free conditions. All experimental procedures were approved by the Institutional Animal Care and Use Committee of the PUMCH (XHDW‐2024‐49). CLP was used to construct a sepsis mouse model, which is described in the Supporting Information. Kaplan–Meier (K‐M) survival curve confirmed the establishment of the CLP mice model (Figure S14). Peripheral blood and spleen were harvested 24 h after modeling.

### 4.3 T Cell Isolation

20 mL fresh peripheral blood was collected within 24 h after enrollment in EDTA‐anticoagulation tubes. CD8^+^ and CD4^+^T cells were sorted by immunomagnetic beads.

### 4.4 Real‐Time PCR Analysis

RNA was extracted using RNeasy Mini Kit (74102, QIAGEN) and reverse transcribed into cDNA (PrimeScript RT Master Mix, RR036A, TaKaRa). The transcription levels of the target genes were quantified using TB Green Premix Ex TaqII (RR820A, TaKaRa). The forward and reverse primers used are listed in Table S3.

### 4.5 Flow Cytometry

For surface staining, 2 × 10^6^/100 µL cells were stained with surface antibody for 20 min at room temperature. After surface staining, cells were washed twice with PBS. Samples were analyzed on BD LSRFortessa (BD Biosciences), and data were analyzed using FlowJo software (TreeStar, USA).

### 4.6 Single‐Cell Data Sequencing and Data Analysis

CD8^+^ and CD4^+^ T cells from 10 patients with septic shock and 10 age‐and sex‐matched HCs were collected for scRNA‐seq using the V2 single‐cell reagent kit (10 × Genomics) and sequenced on Illumina NovaSeq X Plus platform (Shanghai, China). Raw data were processed following the standard Chromium Cell Ranger pipeline software (version 7.0.0) against the GRCh38 human reference genome. Downstream data analysis was conducted using the Seurat package (version 5.0.1) in the R (version 4.3.2) environment, as described previously [60, 61]. The clusterProfiler package (version 4.10.0) was used to conduct the KEGG pathway analysis [62]. The data were visualized using the package ggplot2 (version 3.4.4) in the R environment. We utilized Immunarch (version 0.9.1) and scRepertoire (version 2.2.1) software for the TCR analysis. Paired scTCR‐seq data were integrated with scRNA‐seq based on their matched unique cell barcodes. Bulk transcriptome analysis was described in the Supporting Information.

### 4.7 Clinical Assessment and Definitions

Septic shock was diagnosed according to the Sepsis‐3 criteria as sepsis with persistent hypotension requiring vasopressors to maintain a mean arterial pressure ≥ 65 mmHg, together with a serum lactate > 2 mmol/L despite adequate fluid resuscitation [1]. Immunosuppression was defined as an absolute lymphocyte count below 1000 cells/µL, a commonly used indicator for sepsis‐induced lymphopenia that has been associated with secondary infection and increased mortality [57]. Disease severity was assessed by two widely recognized scoring systems at enrollment. The APACHE II score (range 0–71) integrates 12 acute physiological measurements, age, and chronic health status, with higher scores indicating greater severity of illness and higher predicted hospital mortality [63]. The SOFA score (range 0–24) grades the dysfunction of six organ systems on a 0–4 scale each, including respiratory, coagulation, hepatic, cardiovascular, neurological, and renal systems, with higher scores reflecting more severe organ dysfunction [64]. Serum concentrations of the pro‐inflammatory cytokines IL‐6, IL‐8, TNF‐α, and IFN‐γ and the anti‐inflammatory cytokine IL‐10 were measured as indicators of the systemic inflammatory response. Clinical outcome was defined as survival or death at 28 days after enrollment, and patients were classified accordingly as survivors or non‐survivors.

### 4.8 Statistical Analysis

Graphs and statistical analyses were performed using GraphPad Prism software (version 9.0). Data are presented as mean ± standard deviation (SD). Normal distribution was tested. The student's *t*‐test was used for normally distributed data; otherwise, the Mann–Whitney test was used. Spearman's correlation analysis was conducted. A *p* < 0.05 was considered statistically significant.

## Author Contributions

Xunyao Wu, Bin Du, and Na Cui conceived the study. Xunyao Wu and Yawen Xie performed scRNA‐seq experiments. Xunyao Wu and Yawen Xie performed bioinformatical analysis. Yawen Xie performed cytometric experiments, RT‐PCR, western blot, and ELISA. Yawen Xie and Guoyu Zhao performed animal experiments. Guoyu Zhao, Xianli Lei, and Jiahui Zhang recruited patients and collected blood samples. Xunyao Wu and Yawen Xie wrote the manuscript. Bin Du, and Na Cui supervised the project and reviewed the manuscript. All authors have approved the submitted version.

## Funding

The work was supported by Clinical Research Enhancement Project of Beijing Chaoyang Hospital (No. CYTS2025A03), CAMS Innovation Fund for Medical Sciences (CIFMS) 2023‐I2M‐2‐002 from Chinese Academy of Medical Sciences, National Key R&D Program of China 2022YFC2009803 from Ministry of Science, and Technology of the People's Republic of China, National High Level Hospital Clinical Research Funding (No. 2022‐PUMCH‐B‐126, No. 2022‐PUMCH‐D‐005, No. 2022‐PUMCH‐B‐111), Peking Union Medical College Hospital Young Reserve Talent Development Program (UHB12076).

## Ethics Statement

The study obtained approval from the Ethics Committee of the Peking Union Medical College Hospital (approval no. I‐22PJ1104). All animal experiments were approved by the Institutional Animal Care and Use Committee of the PUMCH (XHDW‐2024‐49). Written informed consent was obtained from all participants.

## Conflicts of Interest

The authors declare no conflicts of interest.

## Supporting information




**Supporting File 1**: Mco270927‐sup‐0001‐SuppMat.docx

## Data Availability

The raw sequence data have been deposited in the Genome Sequence Archive (Genomics, Proteomics & Bioinformatics 2021) in National Genomics Data Center (Nucleic Acids Res 2022), China National Center for Bioinformation/Beijing Institute of Genomics, Chinese Academy of Sciences (GSA‐Human: HRA009117) that are publicly accessible at https://ngdc.cncb.ac.cn/gsa‐human.
