# Supplementary material for: Restricted TCR Diversity and Expansion of CD177+CD8+ and TLR4+CD4+T Cell Subsets in Septic Shock‐Associated Immunosuppression: A Single‐Cell Multi‐Omics Study
Source: MedComm (2020). 2026 Aug 17;7(9):e70927. doi: 10.1002/mco2.70927 (PMC13478844; doi:10.1002/mco2.70927)
Supplement: Supplementary file 1 [file MCO2-7-e70927-s001.docx]

**Supplementary Information for**

**Restricted TCR Diversity and Expansion of CD177⁺CD8⁺ and TLR4⁺CD4⁺ T Cell Subsets in Septic Shock-Associated Immunosuppression: A Single-Cell Multi-Omics Study**

Xunyao Wu^2#^, Yawen Xie^1#^, Guoyu Zhao^4^, Xianli Lei^4^, Jiahui Zhang^4^, Na Cui^1*^, Bin Du^2,3*^

1 Department of Critical Care Medicine, Beijing Chao-Yang Hospital, Capital Medical University, Beijing, 100020, China

2 State Key Laboratory of Complex Severe and Rare Diseases, Clinical and Science Investigation Institute, Peking Union Medical College Hospital, Chinese Academy of Medical Science and Peking Union Medical College, Beijing 100730, China

3 Medical ICU, Peking Union Medical College Hospital, Peking Union Medical College and Chinese Academy of Medical Sciences, No.1 Shuai Fu Yuan, Beijing100730, China

4 Department of Critical Care Medicine, Peking Union Medical College Hospital, Chinese Academy of Medical Science and Peking Union Medical College, Beijing 100730, China

***** Xun-Yao Wu and Yawen Xie contributed equally to this study.

† Corresponding author

Na Cui, MD, E-mail: [cyyy_cn@163.com](mailto:cyyy_cn@163.com)

Bin Du, MD, E-mail: [dubin98@gmail.com](mailto:dubin98@gmail.com)

**Supplementary materials and methods**

**Healthy candidates’ enrollment**

Age- and sex-matched healthy individuals were enrolled without any of the following conditions: (1) Chronic diseases associated with the circulatory, respiratory, endocrine, or urinary system; (2) autoimmune disease; (3) history of hematological or solid malignancies; (4) acute infection within the last three months. The basic information was compared between health controls and patients in Table S4.

**Magnetic activated cell sorting**

PBMCs were isolated by Ficoll-Hypaque density-gradient centrifugation and labeled with CD4 and CD8 microbeads (130-096-533,130-045-201, Miltenyi Biotec, Germany). CD8^+^ T cells were isolated by positive selection, and CD4^+^ T cells were isolated by negative selection using an LS column and a magnetic bead separator. The viability of isolated CD8^+^ and CD4^+^ T cells was > 90%.

**Antibodies used in flow cytometry**

PE/Cyanine7 anti-human CD45RA (304126, BioLegend), APC/Cyanine7 anti-human CD3 (300318, BioLegend), Brilliant Violet 421 anti-human CD3 (300434, BioLegend), FITC anti-human CD8 (980908, BioLegend), PE anti-human CD177 (315806, BioLegend), APC/Cyanine7 anti-human CD4 (317418, BioLegend), PE anti-human TLR4 (312806, BioLegend), APC/Cyanine7 anti-mouse CD3 (100222, BioLegend), FITC anti-mouse CD4 (100406, BioLegend), Brilliant Violet 421 anti-mouse CD8a (100738, BioLegend), PE/Cyanine7 anti-mouse TLR4 (145407, BioLegend), and Alexa Fluor 647 anti-mouse CD177 (566599, BD Pharmingen).

**Antibodies used in Western Blotting**

The following primary antibodies were used: Interleukin (IL)-17RA Rabbit mAb (12661T, Cell Signaling Technology), c-Fos Monoclonal antibody (66590-1-Ig, Proteintech), S100A8 Polyclonal antibody (15792-1-AP, Proteintech), and S100A9 Polyclonal antibody (26992-1-AP, Proteintech).

**CLP murine model**

Mice were anesthetized with 1.25% tribromoethanol, and their abdomen was shaved and disinfected. Following disinfection, an incision was made in the middle of the abdomen to expose the cecum, which was ligated below the ileocecal valve. The cecum was punctured using a 22-gauge needle to extrude little feces and was later returned to the abdomen. After suturing, the mice were subcutaneously injected with 1 mL of normal saline. The mice in the sham group underwent the same procedure except for ligation and puncture. After 24 h, peripheral blood was obtained from the infraorbital vein, and the mice were euthanized using CO_2_ to harvest the spleen.

**Cell isolation from mouse tissues**

Peripheral blood was stored in heparin lithium-anticoagulant tubes at room temperature for > 30 min and centrifuged at 3500 rpm for 5 min to obtain plasma. Then, 5 mL red blood cell lysis buffer was mixed with blood and incubated for 20 min. After washing with PBS and centrifuging, the cells were subjected to flow cytometry analysis.

After euthanasia, the spleen was harvested and ground in a 6-well plate with PBS. The spleen homogenates were washed using PBS and centrifuged. After discarding the supernatant, 3 mL of red blood cell lysis buffer was used to resuspend the cells, which were then centrifuged for 5 min. The cells were stained after washing with PBS.

**Western blotting**

Cells were lysed with RIPA lysis buffer and mixed with a protease inhibitor cocktail (Beyotime, China). The total protein concentration was quantified using a BCA protein assay kit (ThermoFisher, USA). After separation by SDS-PAGE, the proteins were transferred onto a polyvinylidene difluoride membrane (Millipore, USA). The membrane was blocked with 5% bovine serum albumin for 1 h at room temperature and incubated with primary antibodies overnight at 4 ℃. Subsequently, the membrane was washed thrice and incubated with a horseradish peroxidase-conjugated secondary antibody (1:5000) for 1 h at room temperature. The results were visualized by enhanced chemiluminescence (Peirce), and signals were read and analyzed using Image Lab software (Bio-Rad).

**Enzyme-Linked Immunosorbent Assay (ELISA)**

We used ELISA to quantify the concentration of S100A8, S100A9, and S100A12 in serum (catalog: JL14387, JL19155, and JL19159, Jianglai Biotechnology, China). Serum was added in duplicate to pre-coated 96-well plates and incubated at 37°C for 1 h. After washing, biotinylated detection antibodies and HRP-conjugated streptavidin were sequentially added and incubated. The color reaction was developed with TMB substrate, and absorbance was acquired at 450 nm. Concentrations were calculated from standard curves generated with provided recombinant standards.

**Quality control criteria of single-cell transcriptome**

Cells were recognized as low quality and filtered out with meeting any of the following criteria: (1) nFeature_RNA < 200; (2) the percentage of mitochondrial gene expression > 20%. The DoubletFinder package (version 2.0.3) was used to remove doublets. Samples were merged and the batch effects were addressed by Harmony algorithm.

**Bulk RNA-sequencing and data analysis**

Total RNA was extracted from eight CD8^+^ and CD4^+^ T cells of patients with septic shock and four age- and sex-matched HCs. RNA sequencing was performed on the Illumina NovaSeq X Plus platform (Shanghai, China). The raw reads were processed with Fastp (version 0.23.1) using a bioinformatics pipelinetool. Packages limma (version 3.58.1) was used to analyze the DEGs in the R environment. DEGs were determined by absolute (log2Foldchange) > 2 and adjusted *p*-value < 0.05. Gene set enrichment analysis (GSEA) of the CD8^+^ T cell bulk RNA-seq data was performed using the clusterProfiler package (version 4.10.0)⁵⁶. All expressed genes were ranked by the moderated t-statistic, which was analyzed using the gseKEGG function. A fixed random seed was applied to ensure reproducibility. P values were adjusted for multiple comparisons using the Benjamini–Hochberg method, and pathways with an adjusted P value < 0.05 were considered significantly enriched. The enrichment plot of the IL-17 signaling pathway was generated using the enrichplot package.

**Table S1** Characteristics of patients for sequencing

|  | Total  (n = 10) | scRNA-seq + TCR-seq | | |
| --- | --- | --- | --- | --- |
|  |  | Non-survivor  (n = 5) | Survivor  (n = 5) | P value |
| **Baseline characteristics** | | | | |
| Age | 55±14.8 | 55.2±16.4 | 54.8±15.1 | 0.969 |
| Gender, male | 3 (30%) | 2 (40.0%) | 1 (20.0%) | 1 |
| **Severity at the admission to ICU** | | | | |
| APACHE Ⅱ | 18.2±6.25 | 23.4±3.97 | 13.0±2.12 | 0.002 |
| SOFA | 11.7±3.83 | 14.2±2.86 | 9.20±3.03 | 0.028 |
| **Comorbidities, n (%)** | | | | |
| Chronic heart failure | 2 (20%) | 1 (20.0%) | 1 (20.0%) | 1 |
| COPD | 0 | 0 | 0 | 1 |
| Diabetes mellitus | 5 (50%) | 3 (60.0%) | 2 (40.0%) | 1 |
| Chronic kidney disease | 2 (20%) | 1 (20.0%) | 1 (20.0%) | 1 |
| Chronic liver failure | 1 (10%) | 0 (0.00%) | 1 (20.0%) | 1 |
| **Laboratory tests** | | | | |
| Lactate (mmol/L) | 6.04±0.78 | 6.48±0.52 | 5.60±0.53 | 0.029 |
| Creatinine (μmol/L) | 140.2±79.4 | 188±87.4 | 92.2±27.9 | 0.068 |
| Total bilirubin (μmol/L) | 50.14±40.8 | 51.3±41.9 | 49.0±44.5 | 0.936 |
| Albumin (g/L) | 31.1±5.51 | 27.8±4.76 | 34.4±4.28 | 0.050 |
| PCT (ng/mL) | 6.67±5.58 | 10.2±6.24 | 3.18±0.72 | 0.066 |
| CRP (mg/dL) | 142.8±87.2 | 206±71.5 | 79.8±45.6 | 0.013 |
| **Immune parameters** | | | | |
| Lymphocyte (cells/mm3) | 434±173 | 300±120 | 568±89.3 | 0.005 |
| T cell count (cells/mm3) | 262±111 | 178±71.4 | 346±72.2 | 0.006 |
| CD4+T cell count (cells/mm3) | 162±68.1 | 118±42.8 | 207±60.6 | 0.031 |
| CD8+T cell count (cells/mm3) | 92.3±45.3 | 57.2±31.4 | 127±23.5 | 0.005 |
| CD28+CD4+T cell count (cells/mm3) | 129.5±75.5 | 104±97.1 | 155±42.5 | 0.330 |
| CD28+CD8+T cell count (cells/mm3) | 39.4±28.0 | 28.4±21.6 | 50.4±31.5 | 0.238 |
| **Prognosis** | | | | |
| 28-day mortality, n (%) | 5 (50%) | 5 (100%) | 0 (0%) |  |

**Table S2** Characteristics of patients for validation experiments

|  | Total  (n = 51) | Flowcytometry | | | RT-PCR  (n = 8) | Western Blot  (n = 12) |
| --- | --- | --- | --- | --- | --- | --- |
|  |  | Non-survivor  (n = 25) | Survivor  (n = 26) | P value |  |  |
| **Baseline characteristics** | | | | | | |
| Age | 62.1±15 | 64.2±15.5 | 60.1±14.6 | 0.338 | 58.9±12.8 | 66.5±12.1 |
| Gender, male | 22 (43.1%) | 12 (48.0%) | 10 (38.5%) | 0.686 | 4 (50%) | 7 (58.3%) |
| **Severity at the admission to ICU** | | | | | | |
| APACHE Ⅱ | 16.1±4.7 | 17.6±5.48 | 14.7±3.26 | 0.026 | 15.6±5.37 | 15.9±4.01 |
| SOFA | 10.7±3.7 | 11.8±4.07 | 9.62±2.98 | 0.032 | 11.1±4 | 11±3.92 |
| **Comorbidities, n (%)** | | | | | | |
| Chronic heart failure | 12 (23.5%) | 8 (32.0%) | 4 (15.4%) | 0.285 | 2 (25%) | 3 (25%) |
| COPD | 2 (3.92%) | 1 (4.00%) | 1 (3.85%) | 1 | 0 (0.00%) | 0 (0.00%) |
| Diabetes mellitus | 13 (25.5%) | 5 (20.0%) | 8 (30.8%) | 0.575 | 2 (25%) | 5 (41.7%) |
| Chronic kidney disease | 1 (1.96%) | 1 (4.00%) | 0 (0.00%) | 0.490 | 0 (0.00%) | 0 (0.00%) |
| Chronic liver failure | 0(0.00%) | 0 (0.00%) | 0 (0.00%) |  | 0 (0.00%) | 0 (0.00%) |
| **Laboratory tests** | | | | | | |
| Lactate (mmol/L) | 6.19±0.69 | 6.42±0.58 | 5.97±0.73 | 0.017 | 6.14±0.64 | 6.17±0.51 |
| Creatinine (μmol/L) | 90.6±58.8 | 103±71.5 | 79.1±41.5 | 0.162 | 72.8±33.2 | 85.8±35.7 |
| Total bilirubin (μmol/L) | 33.8±27.5 | 31.6±24.2 | 35.9±30.7 | 0.577 | 40.7±38.3 | 35.5±30.7 |
| Albumin (g/L) | 33.1±4.45 | 32.6±5.09 | 33.6±3.77 | 0.424 | 30±4 | 33.5±5.8 |
| PCT (ng/mL) | 10±11.5 | 13.4±14.8 | 6.68±5.53 | 0.040 | 5.0±4.3 | 9.7±6.4 |
| CRP (mg/dL) | 177±120 | 178±124 | 176±117 | 0.968 | 136.2±39 | 211±48.4 |
| **Immune parameters** | | | | | | |
| Lymphocyte (cells/mm3) | 590±211 | 502±221 | 676±163 | 0.003 | 601±141 | 604±112 |
| T cell count (cells/mm3) | 396±151 | 328±148 | 461±126 | 0.001 | 388±94.5 | 417±82 |
| CD4+T cell count (cells/mm3) | 233±94 | 195±93.9 | 270±79.6 | 0.003 | 222±44.5 | 239±61 |
| CD8+T cell count (cells/mm3) | 149±95.3 | 122±90.9 | 175±93.8 | 0.046 | 154±78 | 157±80.6 |
| CD28+CD4+T cell count (cells/mm3) | 221±93 | 192±99.7 | 250±77.6 | 0.026 | 213±46 | 237±74 |
| CD28+CD8+T cell count (cells/mm3) | 78.7±45 | 62.8±43.5 | 94.0±41.0 | 0.011 | 89±37.6 | 71±28 |
| **Prognosis** | | | | | | |
| 28-day mortality, n (%) | 25 (49.0%) | 25 (100%) | 0 (0.00%) |  | 4 (50%) | 5 (41.7%) |

**Table S3** Primer sequences

| **Gene** | **Forward primer** | **Reverse primer** |
| --- | --- | --- |
| CD177 | GTCTATGGAAGGCTGTCTG | TGAGTGGATGGTGGTCTT |
| CDA | GCCAAGAAGTCAGCCTAC | GTCCGTTCAGCACAGATG |
| MCEMP1 | ATGGTGAAGAATGCTGAGAT | TTATTGAGGTGAGGACTGTG |
| MMP9 | AACCAATCTCACCGACAG | GGCAAGTCTTCCGAGTAG |
| NFE2 | AGAGATGGAACTGACTTGG | GCTTGGAGACTGGTATGG |
| S100A12 | ACCAATACTCAGTTCGGAAG | GACCTGTTCATCTTGATTAGC |
| S100A8 | AATTGCTAGAGACCGAGTG | TATCACCAGAATGAGGAACT |
| S100A9 | ACACCTTCCACCAATACTC | TCAGCATGATGAACTCCTC |
| SLC11A1 | ACCTTCTTCTTCCTCTTCCT | TGTTGGCTTCTCTGATGTC |

**Table S4** Basic information of health controls and septic shock patients

|  | scRNA-seq + TCR-seq | | |  | Validation experiments | | |
| --- | --- | --- | --- | --- | --- | --- | --- |
|  | Controls  (n = 10) | Patients  (n = 10) | P value |  | Controls  (n = 32) | Patients  (n = 10) | P value |
| Age | 53.9±13.9 | 55.0±14.8 | 0.866 |  | 56.9±13.5 | 62.1±15.1 | 0.111 |
| Gender, male | 3 (30%) | 3 (30%) | 1 |  | 13 (41.9%) | 22 (43.1%) | 1 |

**Figure S1** Purity of unsorted PBMCs and sorted cells. Unsorted PBMC was gated by FSC and SSC to show CD3^+^CD4^+^T cells and CD3^+^CD8^+^T cells (A). Similar gating strategy was used in sorted CD4^+^T cells (B) and sorted CD8^+^T cells (C).

**Figure S2** TCR repertoire diversity comparison. (A) Comparison of CD4^+^T cells between HCs (n = 10) and septic shock patients (n = 10); (B) Comparison of CD8^+^T cells between HCs (n = 10) and septic shock patients (n = 10); (C) Comparison of CD4^+^T cells between survivors (n = 5) and non-survivors (n = 5); (D) Comparison of CD8^+^T cells between survivors (n = 5) and non-survivors (n = 5).

**Figure S3** TCR clonal expansion characteristic between survivors (n = 5) and non-survivors (n = 5) in (A) CD8^+^ T cells and (B) CD4^+^ T cells.

**Figure S4** TCR repertoire characteristics in different ages. (A) The correlation analysis between age and TCR diversity indicators of CD4^+^T cells (n = 10); (B)The correlation analysis between age and TCR diversity indicators of CD8^+^T cells (n = 10); (C) Comparison of TCR diversity indicators between > 55 years old (n = 5) and ≤55 years old (n = 10) in CD4^+^T cells; (D) Comparison of TCR diversity indicators between > 55 years old (n = 10) and ≤55 years old (n = 10) in CD8^+^T cells; (E) Clonal expansion characteristics between > 55 years old (n = 10) and ≤55 years old (n = 10) in CD4^+^T cells; (F) Clonal expansion characteristics between > 55 years old (n = 10) and ≤55 years old (n = 10) in CD8^+^T cells.

**Figure S5** Comparison of the percentage of CD8^+^T cell subsets between survivors (n = 5) and non-survivors (n = 5).

**Figure S6** (A) The correlation plot of age and CD177^+^CD8^+^ T cell proportion (n = 10); (B) Comparison of CD177^+^CD8^+^ T cell proportion between >55 (n = 5) and ≤55 years old group (n = 5); (C) The correlation plot of age and TLR4^+^CD4^+^ T cell proportion (n = 10); (D) Comparison of TLR4^+^CD4^+^ T cell proportion between >55 (n = 5) and ≤55 years old group (n = 5).

**Figure S7** Comparison of the percentage of CD4^+^T cell subsets between survivors (n = 5) and non-survivors (n = 5).

**Figure S8** Gating strategy of CD8^+^CD177^+^T cells.

**Figure S9** Genome-wide expression distribution in CD8^+^T cells from septic shock patients with CD177 highlighted to mention its overall expression level among all genes.

**Figure S10** Serum level of S100A8, S100A9, S100A12. (A) Comparison on the serum level between HCs (n = 32) and septic shock patients (n = 51); (B) Comparison on the serum level between survivors (n = 26) and non-survivors (n = 25); (C) The correlation between serum levels and transcriptional level in CD8^+^T cells (n = 8).

**Figure S11** Comparison on the proportions of T cell, CD4+T cell, and CD8+T cell (A) between HCs (n = 32) and septic shock patients (n = 51), and (B) between survivors (n = 26) and non-survivors (n = 25).

**Figure S12** Gating strategy of CD4^+^TLR4^+^T cells.

**Figure S13** The correlation between the serum level of S100A8 and S100A9 and their transcriptional levels in CD4^+^T cells (n = 8).

**Figure S14** K-M survival curve of sham (n = 20) and CLP mice (n = 23).
